# The essential role of rehabilitation in operative and non-operative shoulder management: A 40-year experience (1985–2025)

**DOI:** 10.1051/sicotj/2026022

**Published:** 2026-05-13

**Authors:** Dominique F. Gazielly, Marius M. Scarlat

**Affiliations:** 1 Genolier Clinic - Route du Muids 3, 1272 Genolier, Switzerland and General Hospital of Lozère 48000 Mende France; 2 Clinique Chirurgicale St Michel, Groupe ELSAN Av Orient 83100 Toulon France

**Keywords:** Shoulder, Rehabilitation, Surgery, Functional recovery

## Abstract

*Introduction*: As early as 1985, Charles S. Neer II and Peter Welsh emphasized that successful shoulder treatment – whether surgical or non-surgical – relies on structured rehabilitation based on simple exercises performed independently by the patient several times daily. The Sarah Jackins auto-rehabilitation program, developed with Frederick A. Matsen and Douglas T. Harryman in Seattle, further reinforced this concept and was widely implemented in clinical practice and training. *Methods*: This paper describes the application of four key rehabilitation principles in more than 24,000 patients treated for shoulder conditions, including over 8,000 surgical cases. *Results and Discussion*: The protocol is based on (1) simple self-administered exercises performed three to five times daily, (2) supervision by a trained physiotherapist. The paper is well illustrated with examples of all the exercises performed. *Type of paper*: Descriptive, Level V of evidence, Expert Opinion.

## Introduction

In the 1980s, surgical treatment for subacromial impingement, rotator cuff tears, and shoulder arthroplasty remained relatively uncommon in France and across Europe.

The Lyon school, led by Professor Albert Trillat, supported and encouraged one of the authors (DFG) to undertake fellowships with Peter Welsh in Toronto and Charles S. Neer II in New York [1]. In the 1990s, a similar opportunity was offered to MMS, who trained with Frederick A. Matsen, Douglas T. Harryman, and Kevin Smith at the University of Washington in Seattle.

During these experiences, we were struck by the excellent functional outcomes observed in operated patients. At that time, surgery was performed exclusively using open techniques, with arthroscopy emerging in the 1990s. Patients were typically pain-free, demonstrated full range of motion, and returned to professional and sporting activities with a high level of satisfaction.

These reproducible results appeared to depend on two key factors. First, careful and tissue-preserving surgical techniques, using minimally invasive approaches, allowed for reduced postoperative pain and non-restrictive immobilization. Second, and more importantly, the implementation of a structured pre- and postoperative rehabilitation protocol based on simple exercises taught to the patient before surgery and performed independently several times per day.

Postoperative rehabilitation included early passive mobilization, followed by assisted active motion and progressive strengthening, enabling rapid and reproducible functional recovery. Upon returning to France, we adopted and adapted this rehabilitation protocol, incorporating systematic supervision and monitoring by physiotherapists specifically trained in shoulder rehabilitation. This protocol has since been consistently applied in our clinical practice and is presented in this study [1, 2].

## Methods

### Rehabilitation protocol: principles

Largely inspired by the Neer protocol, our rehabilitation approach is applied to both operated and non-operated patients. It is indicated for painful and stiff shoulders, irreparable rotator cuff tears, and postoperative management. Preoperative rehabilitation plays a critical role in facilitating postoperative recovery [3].

The exercises are simple and require no specialized equipment. They are performed as self-rehabilitation, with regular supervision by a physiotherapist trained in shoulder management. Physiotherapy sessions typically last approximately 20 minutes and are conducted without the use of machines or pulleys. Each session begins and ends with manual therapy, including massage of the shoulder girdle and cervical spine.

The physiotherapist plays a central role in the rehabilitation process. Beyond manual treatment, they guide the patient, build confidence, ensure correct execution of exercises, and monitor adherence to the self-rehabilitation program. They are also responsible for identifying potential complications, such as excessive pain, stiffness, or wound abnormalities, and must be able to communicate directly with the treating surgeon when necessary.

Patients perform self-rehabilitation exercises three to four times daily, while supervised physiotherapy sessions are conducted two to three times per week. The physiotherapist thus acts as a key intermediary between the patient and the surgeon, ensuring optimal progression and adaptation of the rehabilitation program.

### Passive mobilization

Passive mobilization exercises represent a clinical application of the concept of Continuous Passive Motion (CPM), introduced by Robert Salter [4, 5]. This concept is based on the principle that passive joint movement stimulates synovial fluid production, thereby promoting cartilage nutrition and preventing stiffness associated with prolonged immobilization.

These exercises are performed both with the physiotherapist and independently by the patient as part of the self-rehabilitation program. Their primary objective is to prevent postoperative stiffness, a major source of pain and functional limitation. Passive mobilization is systematically combined with light, removable immobilization, reduced to a minimum, and initiated immediately after surgery.

This approach is applied following arthroscopic procedures, shoulder arthroplasty, coracoid transfer, and osteosynthesis of proximal humeral fractures. Regular passive mobilization is also a cornerstone in the treatment of painful, stiff shoulders, and adhesive capsulitis.

**Figure 1:**
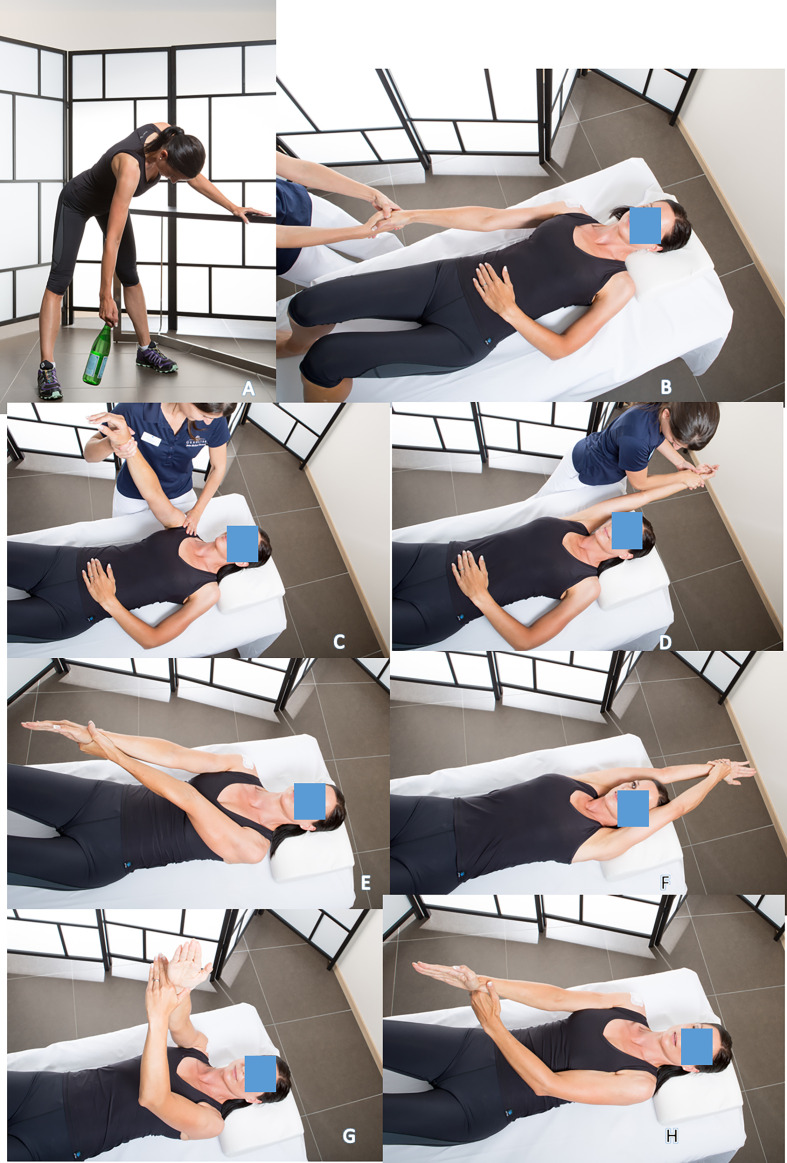
Passive flexion with the physiotherapist, in a lying position on the back, after an analgesic decoaptation, (1A) the physiotherapist pulls the arm until passive flexion obtained (1B), rehabilitation with assistance and self-rehabilitation 3 to 5 times/day (1C, 1D, 1E, 1F). The elbow is kept extended and the hand on the affected side must fight forcefully against the resistance of the hand on the healthy side (1G, 1H).

Each session begins with a massage followed by a warm-up with pendulum exercises, called “Shoulder Aspirin” by DFG in the 1990’ (Figure 1A). A 2-kilogram weight is sometimes used to passively distract the shoulder.

Passive flexion with the physical therapist, in a lying position on the back, knees bent. After a “pumping” (Figure 1B) of analgesic decoaptation of the shoulder, the physiotherapist exerts strong traction on the arm until complete passive flexion is obtained (Figures 1C–1D). The same type of exercise is performed by the patient alone, in self-rehabilitation, 3–4 times a day (Figures 1E–1H). For the return to be painless, the elbow must be kept extended and the hand on the affected side must fight forcefully against the resistance of the hand on the healthy side (Figures 1G, 1H).

**Figure 2:**
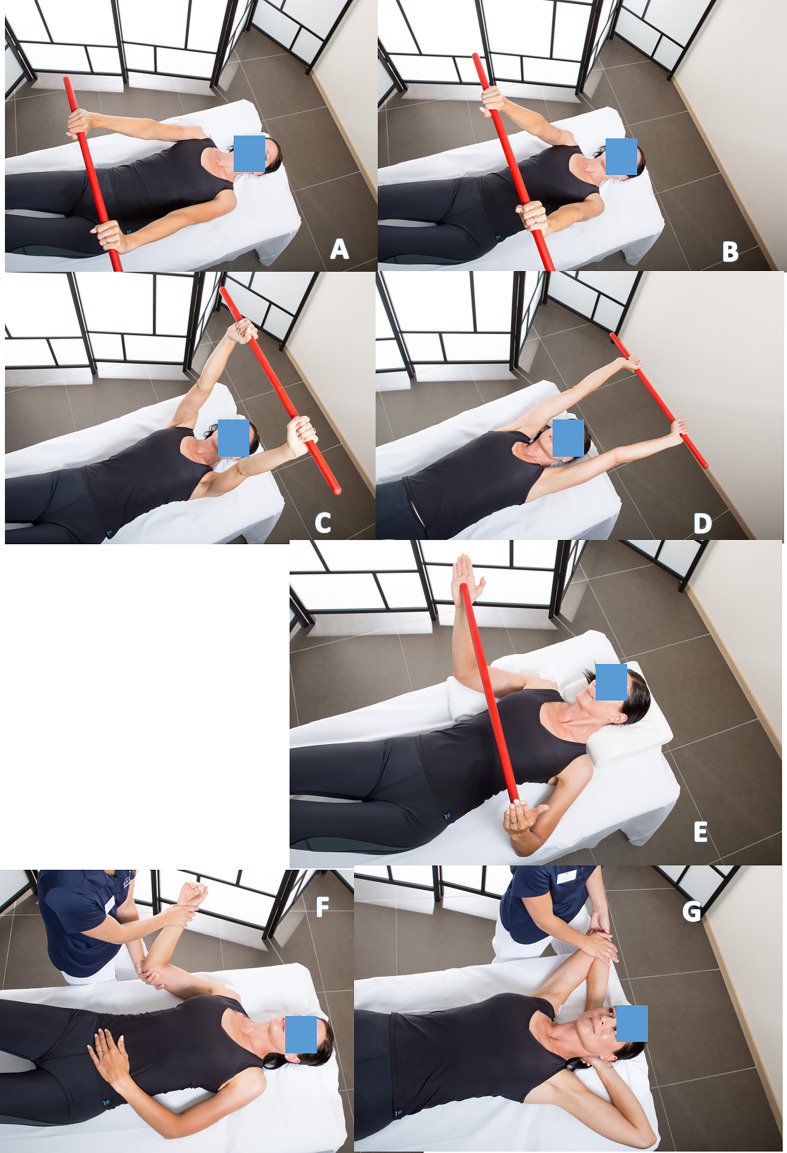
Self-rehabilitation of passive flexion with a stick (2A, 2B, 2C, 2D, 2E). Passive external rotation with the physiotherapist, (2F). External rotation in abduction, called the “nap position” (Figure 2G), opens the antero-inferior capsular pouch of the shoulder (Axillary pouch).

**Figure 3:**
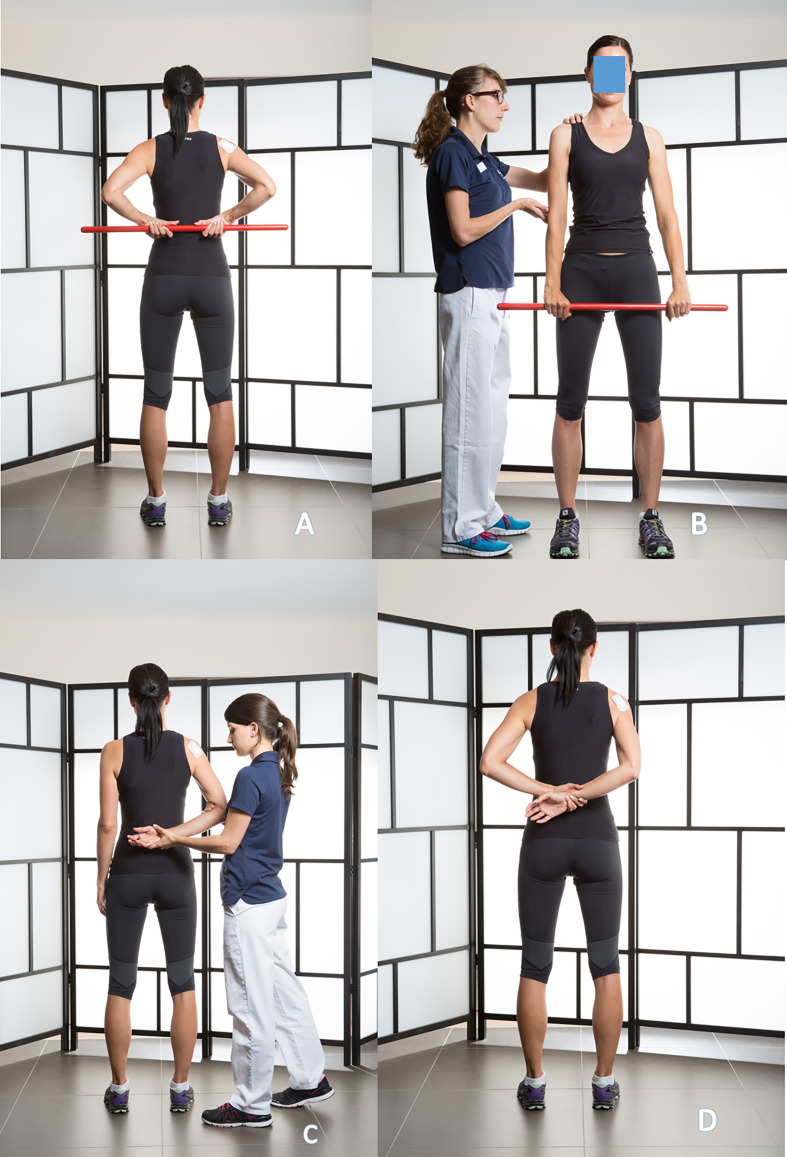
Passive mobilization in internal rotation with physiotherapist (3A), or in self-rehabilitation with a stick (3B), or with the contralateral hand (3C).

- Self-rehabilitation of passive flexion can also be done with a stick (Figures 2A–2D), as external rotation (Figure 2E).
- Passive external rotation is performed with the physiotherapist, lying on the back, knees bent, in slight abduction, with traction-decoaptation downwards (Figure 2F). External rotation in abduction, called “the nap position” (Figure 2G) is essential because it opens the antero-inferior capsular pouch.
- Passive internal rotation in a standing position with the physiotherapist who must ensure to correct a compensatory vicious attitude of the patient, but leaning forward and ascension of the shoulder stump. The rise of the hand must be progressive, gentle and firm (Figure 3A). This exercise is prohibited during the first 3 weeks after a repair of the supraspinatus tendon which would then be put under tension. Passive mobilization in internal rotation can be done either with a stick (Figure 3B), or with the contralateral hand (Figure 3C). The torso should be straight, with a slight adduction of the shoulder blades.

### Assisted active mobilization exercises

Assisted active mobilization exercises are initiated only after full recovery of the passive range of motion. They should systematically be preceded, within the same session, by passive mobilization exercises.

The timing of initiation depends on the surgical procedure. On average, these exercises are introduced 3 weeks after subacromial decompression, 6 weeks after arthroscopic rotator cuff repair [6], and may begin earlier following shoulder arthroplasty, in parallel with passive mobilization [4, 5, 7].

The organization of rehabilitation remains consistent: patients perform self-rehabilitation 3–4 times daily, complemented by two to three supervised sessions per week with a physiotherapist trained in the protocol.

**Figure 4:**
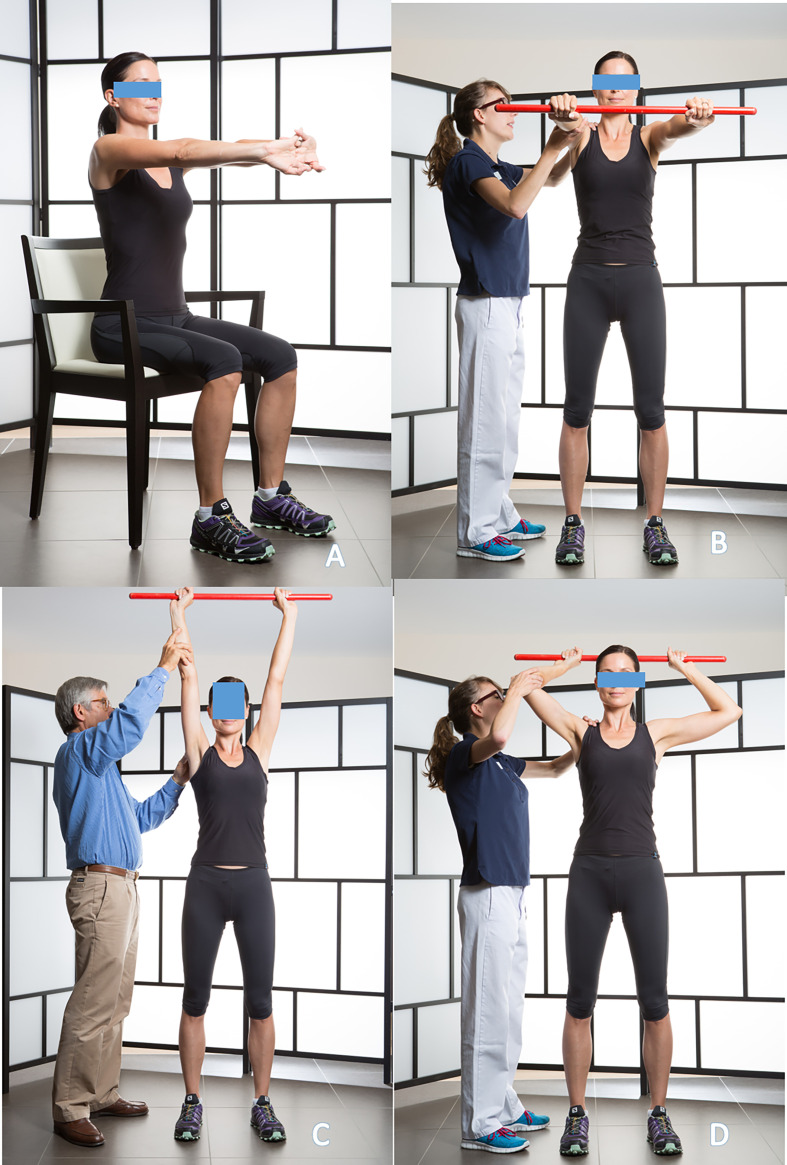
Assisted active flexion with a stick in front of a mirror. Pushing strongly downwards, both elbows straight. (4A). Second step, pushing strongly forward and maintaining the shoulder down at the horizontal (4B). Pushing strongly to zenith (4C). Stick on the head (4D).

Active-assisted forward elevation with a stick is a key exercise, though it is often difficult for patients to perform correctly. Initially, the patient depresses the shoulder girdle by pushing the stick downward with both elbows extended and the trunk upright. Elevation to the horizontal plane is then achieved with assistance from the contralateral arm. This position is maintained while the physiotherapist ensures that the shoulder does not elevate – a common compensatory mechanism associated with subacromial impingement and pain (Figures 4A–4C).

It is advisable to perform this exercise in front of a mirror, with the physical therapist and alone at home, to correct the major defect which is the ascension of the shoulder stump. It is necessary to do two or three sets of ten movements.

The correct postoperative execution of active flexion aided by a more or less stressed patient will be all the easier if the movement has been learned and corrected preoperatively.

Active external rotation, aided in abduction with a stick (Figure 4D). This exercise is essential to prevent any antero-inferior capsular retraction. At the end of the complete assisted active anterior elevation, the patient alone in self-rehabilitation or helped by the physical therapist, places the stick on the head then behind the head, several times, spreading the elbows well.

**Figure 5:**
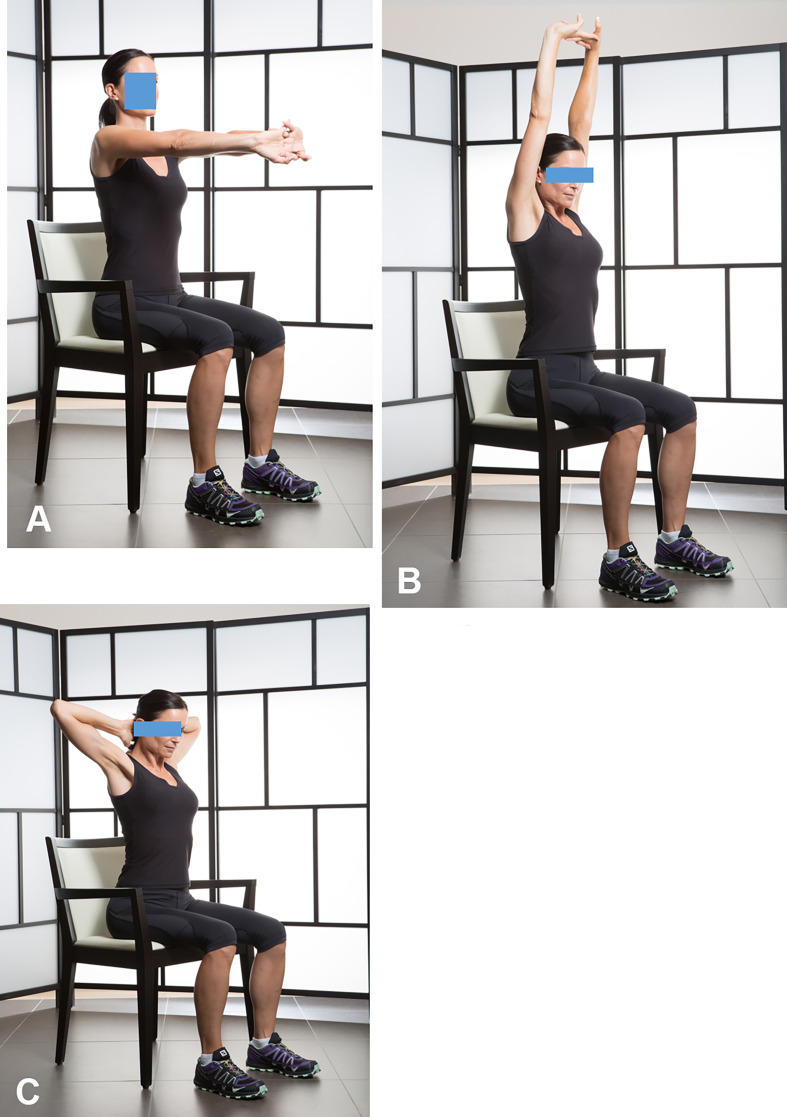
Active elevation and external rotation with abduction (5A); Complete active elevation (5B); Complete active external rotation and abduction (5C).

- Combined active mobility exercise (Figures 5A, 5B). It can be done alone by the patient, at home or in the office, 6 weeks after arthroscopic acromial decompression or 3 months after arthroscopic rotator cuff repair or prosthetic shoulder arthroplasty. It combines active anterior elevation and external rotation in abduction.
- Assisted active internal rotation, hand behind the back, is continued as before (Figures 3A–3C).

### Strength training exercises

Strengthening exercises are initiated only after full recovery of the active range of motion. They should never be performed on a stiff or painful shoulder. These exercises are taught to the patient and performed as part of daily self-rehabilitation, typically twice per day, in addition to weekly supervised sessions with the physiotherapist. The rehabilitation strategy is based on a simplified biomechanical model of the shoulder. Physiological arm elevation results from the balance between the upward force of the deltoid and the downward stabilizing forces of the humeral head depressor muscles. The resulting force vector, aligned with the supraspinatus tendon, allows smooth and coordinated elevation.

**Figure 6:**
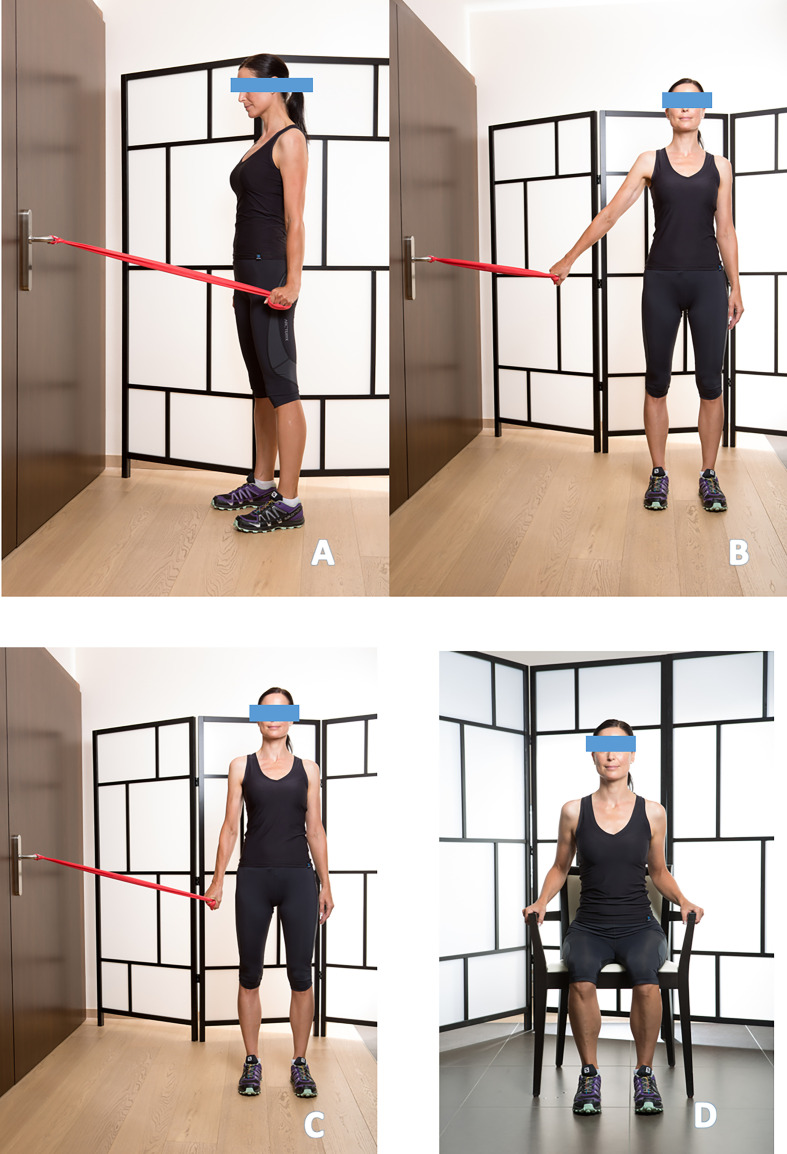
Anterior deltoid rehabilitation (6A); Training of the great pectoralis, elbow extended, mobilization stars at 45° arm abduction (6B); Pectoralis strengthening with isometric hold five seconds (6C); Latissimus dorsi strengthening seated with isometric contractions (6D).

Strengthening focuses on restoring this balance and includes the following exercises (typically five sets of ten repetitions):

**Anterior deltoid strengthening** using elastic resistance bands (Figure 6A).

**Pectoralis major strengthening**, contributes to humeral head depression. The movement should begin at approximately 45° of abduction, with the elbow extended, and be held for five seconds (Figures 6B, 6C).

**Figure 7:**
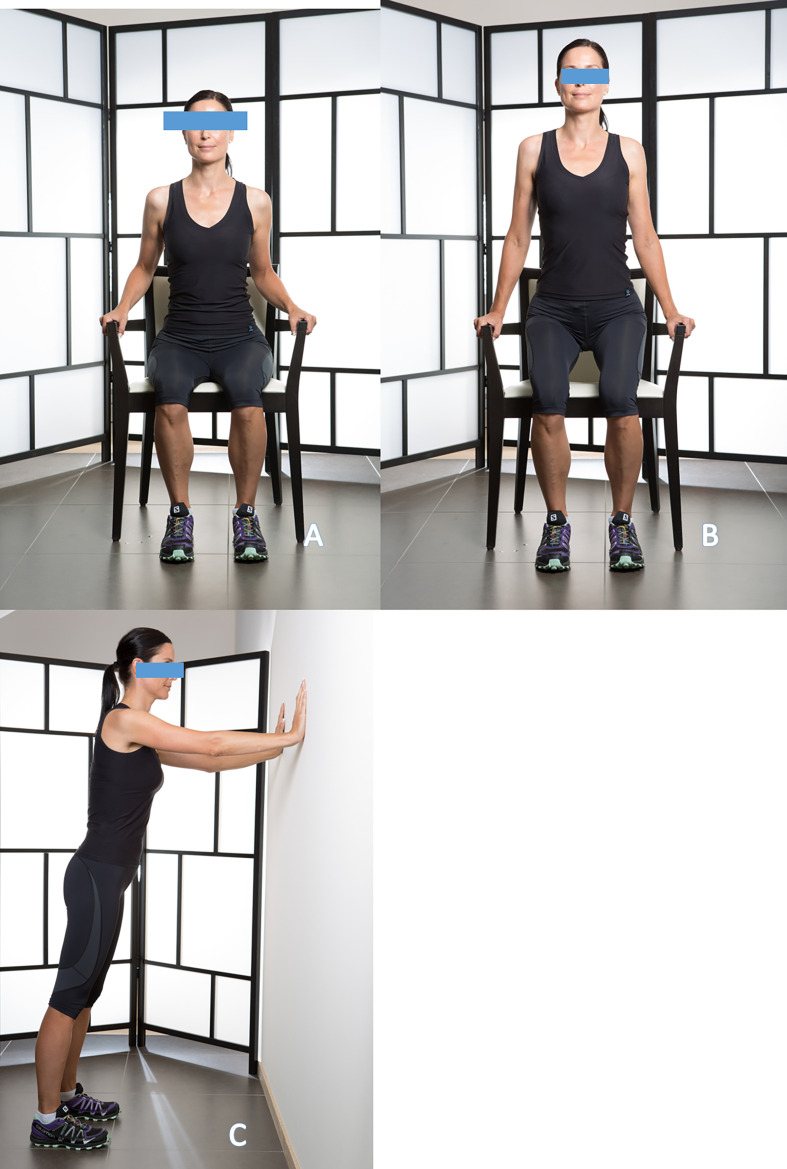
Latissimus Dorsi strengthening seated and standing with push-ups (7A to 7C).

**Latissimus dorsi strengthening**, also acts as a humeral head depressor. Care must be taken to maintain an upright trunk throughout the movement (Figures 7A, 7B).

**Serratus anterior strengthening**, essential for scapular stabilization. Wall push-up exercises are particularly effective (Figure 7C).

Internal and external rotator cuff exercises with an elastic band, in self-rehabilitation, are often poorly performed and can cause pain in the long biceps tendon. They should not be prescribed systematically and recommended in the treatment of non-operated and operated anterior instability, particularly in patients with hyperlaxity. They are performed isometrically.

## Rehabilitation protocol for non-operated patients

Two out of three patients have been treated conservatively since 1985, based on the described rehabilitation protocol.

### Treatment of the stiff shoulder in flexion

Thousands of patients suffer in all the gestures of daily life and especially at night when they sleep on their side. The clinical examination reveals a painful limitation of the anterior elevation (average up to 130°), and of the internal rotation of the hand in the back, limited to the hand-buttock, the external rotation can be severely reduced. This painful stiffness is linked either to a tenosynovitis of the long biceps which “sticks” to the bicipital bone groove, or to a true acromial bone conflict, or to a post-traumatic or postoperative too strict and too long immobilization elbow to the body. Often overlooked, this clinical picture can develop into a true adhesive capsulitis. Recovery of joint ranges of motion is achieved on average in three months if the protocol is followed regularly and the patient is motivated [6, 8–10].

Systematic magnetic resonance imaging (MRI) can demonstrate subacromial bursitis requiring infiltration under ultrasound control. If there is a significant acromial bone spur, arthroscopic subacromial bone decompression could be proposed.

This clinical picture can also be seen in a context of calcific tendinopathy of the cuff diagnosed by a standard radiographic assessment. After infiltration under ultrasound control, a protocol of passive then active assisted mobilization is applied. Strength training exercises, ultrasound and shock waves should be avoided because they are useless and very painful. If the calcium deposit does not disappear spontaneously (90% of cases) and the pain persists despite the infiltrations, arthroscopic excision is proposed.

### Conservative treatment of non-operated full-thickness rotator cuff tears

It is intended for elderly patients, with ruptured tendons, of poor quality making the postoperative functional results uncertain, or with massive ruptures of two or even three tendons retracted to the glenoid, or finally with medical contraindications for reverse arthroplasty. In all cases, the three types of successive exercises are indicated: passive, active assisted and strength training with a trained physical therapist and a motivated patient in self-rehabilitation three times a day.

- In the first case, the patient presents with a clinical picture of a painful and stiff shoulder. The MRI specifies the existence of an acromial conflict, the extent of the rupture, the quality of the remaining tendons and the degree of fatty muscle degeneration. A protocol of passive mobilization, then active assisted, and finally strength training of the depressors can make a functional and painless shoulder. However, arthroscopic smoothening may be indicated, without repair of the cuff but associated with tenotomy of a very degenerative long biceps tendon, if the pain persists after recovery of mobility [9].
- In the second case of massive and irreparable rupture of the cuff, there may be a clinical picture of a pseudo-paralytic shoulder with the inability to actively raise the arm. Before proposing the placement of a reverse shoulder prosthesis, which is the current trend, it is advisable to attempt conservative treatment, well organized with a competent physiotherapist and a motivated patient. The basic biomechanical principle is that the resultant R, instead of being horizontal, has become vertical due to the disappearance of the rotator cuff, and the predominance of the upward force of the Deltoid over the force of the extrinsic depressors A of the humeral head. The humeral head comes to abut under the acromion; active anterior elevation is impossible, giving a picture of a pseudo-paralytic shoulder. The goal of rehabilitation is to reestablish a balance between the forces D and A, and to create an artificial resultant R whose path will have become horizontal again, making anterior elevation possible. After recovery of passive joint amplitudes, priority is given to assisted active mobility exercises (Figures 3–5) in conjunction with strength training exercises (Figures 6, 7). With this protocol, we obtained 82.5% satisfactory results, eliminating the need for reverse shoulder arthroplasty.

## Rehabilitation protocol for operated patients

### Degenerative full-thickness rotator cuff tears

**From October 1986 we repaired rotator cuff tears by open techniques** with procedures learned in North America [1] using minimally invasive approaches and associated with anterior acromioplasty. The suture was protected postoperatively by a removable light splint with 30° abduction. From the beginning, two principles were applied: preoperative rehabilitation and early postoperative mobilization [2, 4, 5].

Active mobility was recovered at the end of the third postoperative month and professional and sports functional recovery within an average of 6 months postoperatively, based on a series of 100 cases, reviewed with a minimum follow-up of 24 months [2].

After 1992, we began repairing full-thickness rotator cuff tears by the arthroscopic technique, learned from Harvard Ellman and Stephen Snyder in Los Angeles, and Richard Caspari in Charleston. The patients received the same protocol as that used for open repairs: immobilization in 30° abduction for 6 weeks, removable at night, early passive mobilization without limitation, except for internal rotation with the hand behind the back for 6 weeks, then assisted active mobilization from the 6th week to the 3rd month postoperatively, then strength training of the depressors of the numeral head. Functional recovery times were comparable to those of an open repair [9].

**Figure 8:**
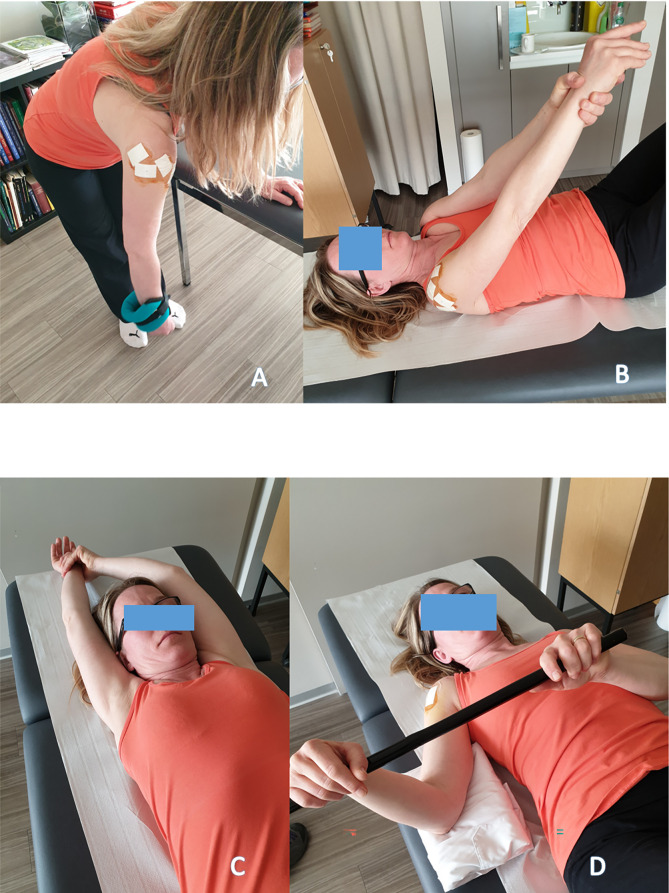
Immediate post-operative passive pendulum exercises, with or without weight (8A); Passive forward elevation controlled with the opposite arm (8B); Passive forward elevation helped with the opposite arm, 140° after cuff repair or shoulder arthroplasty, as tolerated to maximum after subacromial surgery or after capsular release (8C); Post-operative passive external rotation (8D).

**Figure 9:**
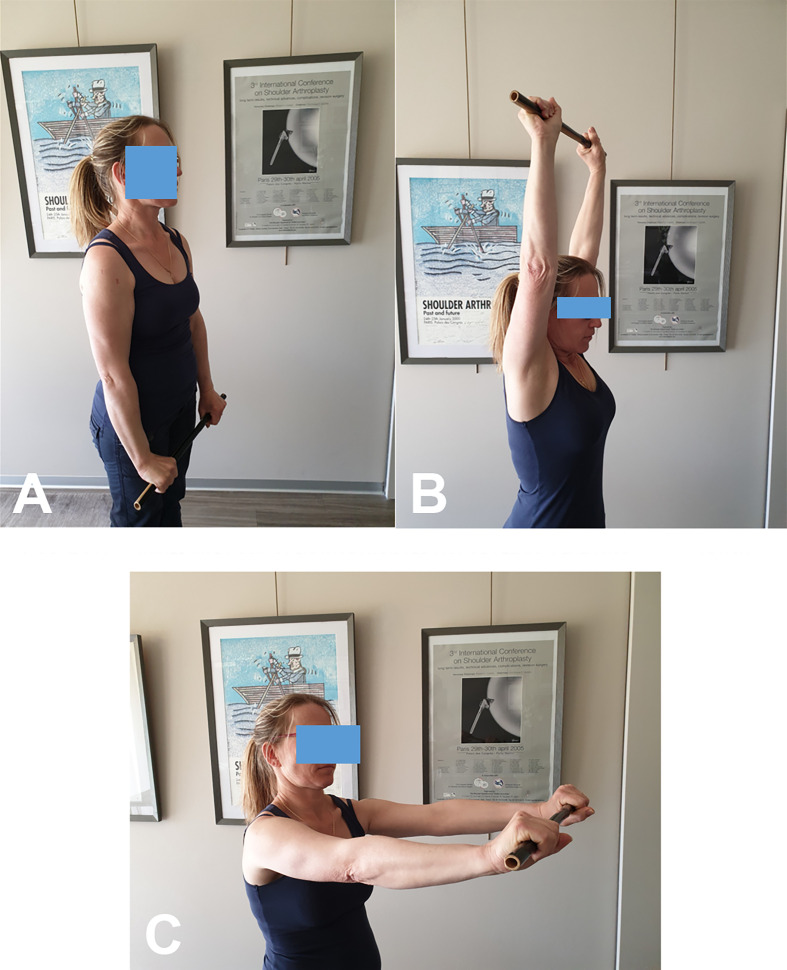
Immediate post-operative assisted active flexion exercises, with a stick (9A and 9B); Assisted active flexion with a stick (9C).

**Figure 10:**
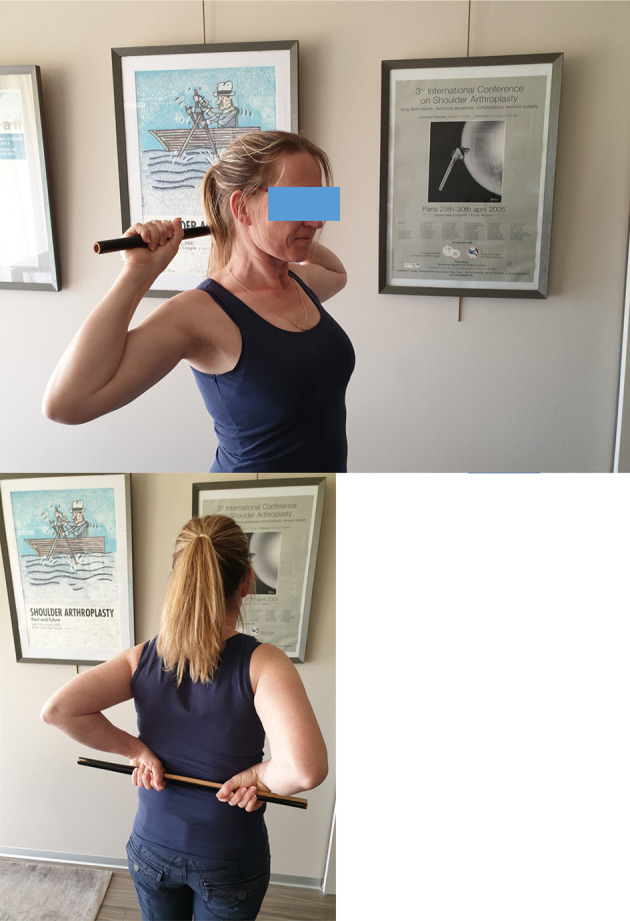
Assisted active external and internal rotation with a stick.

Today, immediate postoperative passive mobilization without limitation is authorized. Pendular exercises (Figure 8A), complete flexion helped with the contralateral arm (Figures 8B, 8C), external rotation with the elbow to the body and helped with a stick (Figure 8C). Assisted active mobilization begins at the 6th postoperative week (Figures 9A–9C), and muscle training of the extrinsic depressors of the humeral head after the 3rd postoperative month (Figures 10A–10B).

### Rehabilitation of patients with anatomic total shoulder arthroplasty

Peter Welsh in Toronto and Charles Neer II in New York taught us the surgical technique of placing a sliding total shoulder prosthesis, according to Neer’s postoperative rehabilitation protocol, focused on immediate postoperative mobilization [4, 5]. The deltopectoral approach, respect for the soft tissues, the meticulous placement of the humeral and especially glenoid implants, care taken in suturing the subscapularis tendon, and a true anterior access to the shoulder – allow immediate postoperative passive mobilization and explain the quality of the functional results obtained: shoulder arthroplasty surgery respects the soft tissues. In the 1990s, we adopted the concept of anatomical prosthesis developed by P. Boileau and G. Walch and used the Aequalis implants.

**Figure 11:**
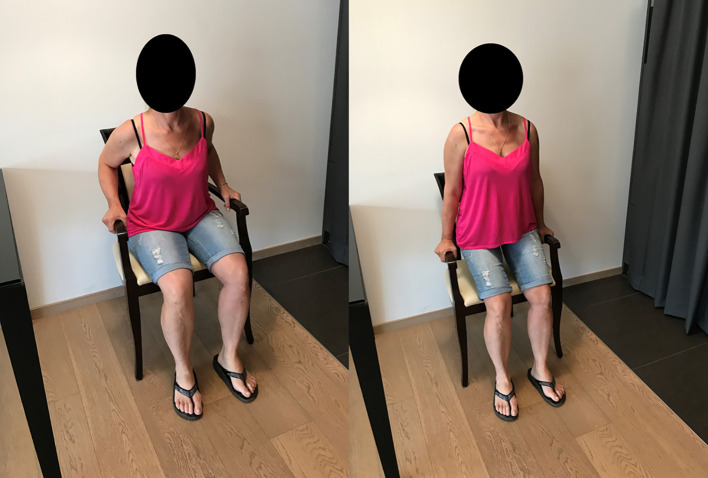
Strengthening of the back with exercises on a chair.

Since 1986, we have applied these principles: meticulous placement technique, respect for the soft tissues, and early postoperative mobilization. The postoperative rehabilitation protocol used (Figures 11A–11B) is based on the same principles: learning exercises preoperatively, light and removable immobilization, not mandatory at night, immediate postoperative passive mobilization in flexion, external and internal rotation, three times a week with the physiotherapist and 3–4 times a day by the patient in self-rehabilitation, active mobilization assisted with a stick in the first postoperative week, recovery of active mobility at the end of the third postoperative month, then gentle muscle-building exercises. The patient recovers a functional shoulder in the 6th postoperative month.

### The Latarjet-Patte coracoid block in the treatment of anterior shoulder instability

Since 1986, we have been using coracoid transfer in the treatment of recurrent anterior dislocations and stabilization with one screw [7]. We used our rehabilitation protocol starting postoperative passive and active assisted mobilization exercises in flexion, external rotation with the elbow to the body and internal rotation with the hand behind the back. The external rotation in abduction (nap position) was prohibited during the first six postoperative weeks. The immobilization with the elbow to the body has always been non-strict, removable, except at night. The antero-inferior stabilization effect of the humeral head is ensured by a triple antero-inferior locking according to Patte: bony (screwed coracoid transfer), muscular (the short biceps tendon presses the lower third of the subscapularis in abduction-external rotation) and the antero-inferior capsule is tightened (7). The reliability of the fixation of the coracoid stop with a single 4.5 mm cortical screw in compression with a washer and the solidity of the musculo-tendinous sutures, in particular of the subscapularis tendon and capsular with non-absorbable thread, make it possible to mobilize the operated shoulder very quickly and to minimize immobilization of the elbow to the body, however necessary in young sports subjects. The complete recovery of all joint ranges, the absence of complications and postoperative recurrences are due to the combination of a meticulous surgical technique and immediate mobilization allowing a return to contact sports at the end of the fourth postoperative month.

## Discussion

In the 1980s, postoperative rehabilitation of the shoulder was characterized by prolonged immobilization and delayed mobilization, particularly in forward elevation and external rotation. Immobilization with the elbow against the body was often maintained for up to 6 weeks, increasing the risk of postoperative stiffness.

Over time, and similarly to the evolution observed in knee rehabilitation, the duration of immobilization has progressively decreased, while early mobilization has become widely accepted among shoulder specialists.

In our practice, early mobilization was introduced as early as 1986, evolving toward immediate postoperative mobilization in the 2000s. This approach has consistently resulted in faster functional recovery and earlier return to daily and sporting activities.

However, prolonged immobilization remains common in many settings and continues to be associated with delayed recovery and increased stiffness. Early passive mobilization, combined with minimal immobilization, appears to improve both the speed and quality of functional recovery.

Several studies support this approach. Tirefort et al. [8] demonstrated that the absence of postoperative immobilization after rotator cuff repair is associated with improved early mobility and functional outcomes compared to sling immobilization. Similarly, Klintberg and Gunnarsson [9], in a randomized study of 114 patients, reported improved early range of motion in patients undergoing early mobilization, without differences in long-term outcomes or strength.

Denard and Lädermann [10] also reported faster recovery following early mobilization after shoulder arthroplasty.

Beyond technical considerations, patient-related factors play a critical role. Motivation and adherence to self-rehabilitation significantly influence outcomes. Levins et al. [11] demonstrated a strong correlation between patient mental health, engagement in rehabilitation, and final functional results.

Furthermore, the physiotherapist plays a central role in both preoperative preparation and postoperative follow-up, providing valuable feedback to the surgeon and contributing to surgical decision-making. The ability of the patient to actively participate in rehabilitation should be considered when determining surgical indications.

From a health economics perspective, this rehabilitation protocol is cost-effective compared to inpatient rehabilitation programs. However, structured rehabilitation centers may still be necessary in selected cases, such as patients living alone or with limited support.

Ultimately, optimal outcomes rely on close collaboration between the surgeon, physiotherapist, and patient. The physiotherapist acts as a key coordinating element, ensuring adherence, early detection of complications, and continuous communication with the surgical team.

## Conclusion

The rehabilitation protocol described in this study is simple, reproducible, effective, and cost-efficient. It is based on a collaborative model involving an experienced surgeon, a physiotherapist trained in shoulder rehabilitation, and a motivated patient actively engaged in self-rehabilitation.

While surgical technique and appropriate indications remain essential, they do not alone guarantee a successful outcome. In shoulder pathology, rehabilitation plays a central role in determining functional recovery.

This experience, accumulated over four decades, supports the concept that structured, early, and patient-centered rehabilitation is a cornerstone of both operative and non-operative shoulder management.

## Data Availability

Data available with the authors. As this is a descriptive review paper based on clinical experience, no individual patient was included in the study.

## References

[1] Gazielly DF (1985) Compte rendu de voyage. Assemblée générale de la SOFCOT. Rev. Chirurgie. Orthop (Paris) 71, 67 (in French).

[2] Gazielly DF, Godeneche JL (1991) Preoperative management and rehabilitation of the rotator cuff tears. In “Surgical Disorders of Shoulder”, Watson MS Editor. Chap 277. Churchill Livingston, London, 1991.

[3] Hugues M, Neer CS II. (1975) Glenohumeral joint replacement and post-operative rehabilitation. Phys Ther 55, 850–858. 1144524 10.1093/ptj/55.8.850

[4] Neer CS II (1990) Shoulder rehabilitation. In: Neer CS II Editor. Shoulder Reconstruction. Philadelphia: WB. Saunders 1990 (7), 487–533.

[5] Salter RB (1993) Continuous Passive Motion (CPM). A biological concept for the yearling and regeneration of articular cartilage, ligaments, tendons: from the original definition to research and clinical applications. Robert Bruce Salter, Williams and Wilkins, p. 419.

[6] Holzer NA, Cunningham G, Laedermann A, Gazielly DF (2013) Latarjet-Patte triple locking procedure for recurrent anterior instability. Tech Shoulder Elbow Surg 14(3), September 2013.

[7] Tirefor J, Schwitzguedel AJ, Collin P, Nowak A, Plomb-Holmes C, Laedermann A (2019) Postoperative mobilization after superior rotator cuff repair: sling versus non sling: a randomized prospective study. J Bone Joint Surg Am 101(6), 494–503. 30893230 10.2106/JBJS.18.00773

[8] Matsen FA, Lippitt, B (2003) Shoulder surgery principles and procedures. Saunders 2003.

[9] Klintberg IH, Gunnarsson AC (2009) Early loading in physical therapy after full-thickness rotator cuff repair: a prospective randomized pilot study with two years follow up. Clin Rehab 23, 622–638. 10.1177/0269215509102952 19482895

[10] Denard PJ, Lädermann A (2016) Immediate versus delayed passive ranges of motion following total shoulder arthroplasty. J Shoulder Elbow Surg 25(12), 1918–1924. 27727055 10.1016/j.jse.2016.07.032

[11] Levins JG, Dasari SP, Quinlan NJ, Whitson AJ, Matsen FA 3rd, Hsu JE (2024) Anatomic shoulder arthroplasty: the correlation between patient resilience, mental health, and outcome. J Shoulder Elbow Surg 33(6S), S9–S15. 38548096 10.1016/j.jse.2024.03.008

